# Interocular differences in choroidal thickness and circulation in anisomyopic adults and their association with myopia severity

**DOI:** 10.3389/fmed.2025.1641704

**Published:** 2025-07-21

**Authors:** Chong Tang, Fanfan Huang, Rong Hu, Yan Ji, Yu Gao, Kai Shi, Yuanyuan Wu, Wenjuan Wan

**Affiliations:** The First Affiliated Hospital of Chongqing Medical University, Chongqing Key Laboratory of Prevention and Treatment on Major Blinding Diseases, Chongqing Eye Institute, Chongqing Branch (Municipality Division) of National Clinical Research Center for Ocular Diseases, Chongqing, China

**Keywords:** choroidal thickness, choroidal circulation, choriocapillaris, myopia, anisomyopia

## Abstract

**Purpose:**

The study aimed to compare interocular differences in choroidal thickness and circulation between the paired eyes of anisomyopic adults and to further investigate the potential connection between the choroid and myopia severity.

**Methods:**

A total of 120 eyes of 60 anisomyopic adults were included in this observational cross-sectional study. All patients underwent detailed ocular examinations, including spherical equivalent refraction (SER), axial length (AL), and other biometric parameters. Mean choroidal thickness (MCT), choriocapillaris blood flow area (CBFA), choroidal vessel volume (CVV), and choroidal vessel index (CVI) were measured over a 6 mm x 6 mm macular area using swept-source optical coherence tomography (SS-OCTA). Interocular differences in the abovementioned measurements between the paired eyes of the anisomyopic individuals were analyzed.

**Results:**

The mean SER was −5.98 ± 1.72 diopters (D) in the more myopic eyes and −3.38 ± 1.71 D in the less myopic eyes, while the mean interocular difference in SER was −2.60 ± 1.17 D. The more myopic eyes had lower values of MCT, CBFA, CVV, and CVI than the relatively less myopic fellow eyes (all *p* < 0.01). The interocular difference in SER was positively correlated with that in MCT, CVV, and CVI (all *p* < 0.05), and the interocular difference in AL was negatively correlated with that in MCT, CVV, and CVI (all *p* < 0.05). The multiple linear regression model with generalized estimating equations showed that the interocular difference in CVV was significantly associated with the interocular difference in SER (*p* < 0.05).

**Conclusion:**

Choroidal thickness and circulation were found to be reduced in the more myopic eyes of anisomyopic adults. We observed that the greater the interocular difference in SER, the lower the choroidal thickness and circulation, indicating that the choroid is impaired in individuals with myopia and is more likely to be compromised with severe myopia.

## Introduction

Myopia is an important global public health issue and can lead to significant vision impairment (1–3). Anisomyopia, a condition related to myopia, is defined as an unequal refractive error between the right and left eyes. This is characterized by an interocular difference in myopic spherical equivalent refraction (SER) ≥ 1.00 D (4). This unequal state of anisomyopia is typically caused by one eye experiencing accelerated growth compared to the contralateral eye during myopia development (5–8). Anisomyopia is significantly prevalent, and its incidence rate has been confirmed to be relatively high, approximately 2 to 5 times higher than that of hyperopic anisometropia (9). Severe anisomyopia is a causative factor for a range of significant visual impairments, including visual fatigue, diplopia, declined stereovision, amblyopia, and strabismus, among others (10, 11).

To date, the pathophysiology of myopia, which is the root cause of the condition of anisomyopia, remains incompletely understood. The majority of previous studies have primarily focused on ocular length, corneal curvature, and anterior chamber depth (ACD) (12–14). However, relying on these downstream indicators alone cannot fully reflect the rapid progression of myopia. Regarding fundus structures, although studies have reached a certain degree of consensus that the retina is impaired as myopia progresses (11, 15, 16), the limited detection techniques have confined fundus research only to the retina, leaving the exploration of the choroid largely untapped. The choroid, as a highly vascularized tissue, the integrity of the structure and function of the choroid are crucial for maintaining retinal homeostasis; any malfunction or malformation in the choroid can be indicative of myopic onset and progression (17–19). Recently, it has been observed that there is already a significant decrease in choroid thickness (ChT) during the initial phase of myopia, even while the retina remains stable (20). Furthermore, another study reported that abnormal variations in choroidal tissue appeared in high myopia before the occurrence of retinopathy (21, 22). Moreover, Ho et al. (23) pointed out that decreased visual acuity in myopic patients was only related to choroid changes in the macular fovea. All these compelling findings strongly imply that the involvement of choroidal mechanisms may contribute to myopia, offering us a novel perspective and guiding myopia research in a new direction. Therefore, we confidently speculate that alterations in the choroid may serve as a potential indicator of myopia. Currently, there are few studies focused on the choroid in anisomyopia. In fact, research on anisomyopia is urgently needed because examining and comparing both eyes of the same individual can minimize the influence of confounding inter-individual variables such as genetics and environment. These natural experimental paradigms can prompt a deeper comprehension of how human myopia develops.

Moreover, previous medical practice has faced challenges in visualizing the choroid using fluorescein angiography (FA) due to obstructions caused by the pigment in the layer of the retinal pigment epithelium and choroid. Traditional imaging modality, indocyanine green angiography (ICGA), allows for the exploration of the choroid, but it is restricted to generating two-dimensional (2D) images of large vessels (17, 24). OCTA is a reliable *in vivo* quasi-histological imaging technology that offers detailed information and quantitative evaluations without the use of dyes or physical contact. Particularly, recent advancements in swept-source optical coherence tomography (SS-OCTA) represent a significant milestone. This technology enables 3D visualization and is equipped with higher frequency, faster scanning speed, deeper signal penetration, and better sensitivity, allowing for unprecedented, optimal visualization of the choroid (7, 24, 25). Furthermore, to the best of our knowledge, anisomyopia has not been extensively observed using OCTA to date. Thus, it is imperative to obtain precise measurements of choroidal circulation and thickness changes using SS-OCTA to investigate the interocular differences between the paired eyes of anisomyopic adults, which will aid in revealing the underlying connection between the choroid and myopia.

## Methods

### Subjects

The First Affiliated Hospital of Chongqing Medical University (CQMU) approved our study, and all subjects provided written informed consent. The study protocol adhered to the tenets of the Declaration of Helsinki.

A total of 60 subjects aged 18–24 years who sought vision correction at the Ophthalmology Clinic in the First Affiliated Hospital of CQMU from August 2023 to November 2023 were enrolled. The inclusion criteria required that all participants were anisomyopic adults with an interocular difference in mean spherical equivalent refraction (SER) of ≥1.0 diopters (D). Binocular best-corrected visual acuity (BCVA) had to be no worse than 1.0, with good fixation function. Intraocular pressure (IOP) had to be normal (10–21 mmHg), and fundus examination had to show no obvious abnormalities. The SS-OCTA image signal intensity had to be greater than or equal to 8. The exclusion criteria were as follows: existing evidence of ocular diseases, such as glaucoma and cataracts; a history of ocular diseases, trauma, or surgery; any diseases that may impact ocular circulation, such as hypertension and diabetes; autoimmune or connective tissue diseases, such as rheumatism; use of drugs that affect vascular function within the past 2 weeks; image signal intensity < 8, and the presence of noticeable center shifts or scan artifacts.

### Ocular biometric measurements

After acquiring a detailed systemic and ocular history from all participants, comprehensive ocular examinations were performed, including measurements of refractive error (using both computer refractometry and subjective refractometry) Prior to these measurements, participants’ pupils were dilated to ≥ 6 mm with compound tropicamide eye drops, and the light reflex was absent. Refractive error was converted to SER by adding half of the cylindrical diopter to the spherical diopter. BCVA was assessed based on the Snellen chart. Additional measurements included IOP (CT-80, Topcon, Japan), axial length (AL), corneal thickness (CCT), corneal curvature radius (CCR), and central anterior chamber depth (ACD) (Pentacam® AXL panoramic biometer, Oculus GmbH, Wetzlar, Germany). A slit lamp anterior segment examination and a fundus examination with a non-contact lens after pupil dilatation were also performed.

### SS-OCTA image extraction

The macula area was scanned and imaged using an SS-OCTA device (VG200S; SVision Imaging, Henan China). This instrument contained an A-line speed of 200,000 A-scans per second, an axial resolution of 5 μm, a lateral resolution of 13 μm, a central wavelength of 1,050 nm, a spectral width in the range of 990–1,100 nm, and a scan depth of 3 mm (26–28). Volume data from fundus images were collected using the Angio 1,024 × 1,024 R4 scan mode. This mode covered a region of 6 mm x 6 mm centered on the macular fovea. The eye-tracking function, designed to eliminate eye-motion artifacts, was enabled during the inspection, and the final image with a signal intensity greater than or equal to 8 (on a scale of 10) was selected.

**Figure 1:**
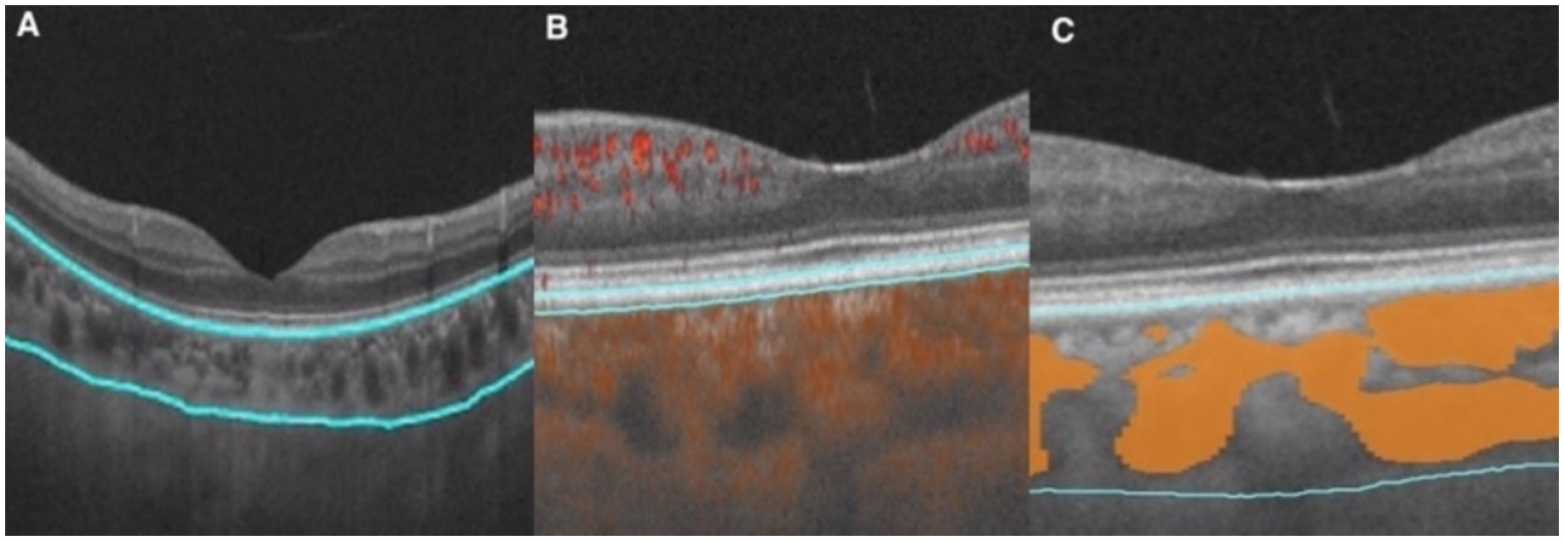
Illustration of each layer of the choroid. **(A)** Choroid layer (the area between the two blue segmentation curves). **(B)** Choriocapillaris layer (the area between the two blue segmentation curves). **(C)** Large and medium vascular volume of the choroid (represented by the orange area).

The choroid is situated at the region extending from the RPE–Bruch’s membrane complex to the choroid–sclera interface, and the choriocapillaris layer is identified as the area between the basal boundary of the RPE-Bruch’s membrane complex and 20 μm below it. The area located 20 μm below the Bruch’s membrane to the choroid-scleral boundary consists of the large and middle choroidal vascular layers (6, 7) (Figure 1). The main outcome measures were mean choroidal thickness (MCT), choriocapillaris blood flow area (CBFA), choroidal vessel volume (CVV), and choroidal vessel index (CVI). CVV was defined as the large and medium vascular volume of the choroid, and CVI is defined as the ratio of CVV to the total choroidal volume (29). All parameters were directly derived from the built-in algorithms of the device, and all examinations were performed by an expert operator from 8:30 AM to 11:30 AM to reduce the potential effects of diurnal variations.

**Figure 2:**
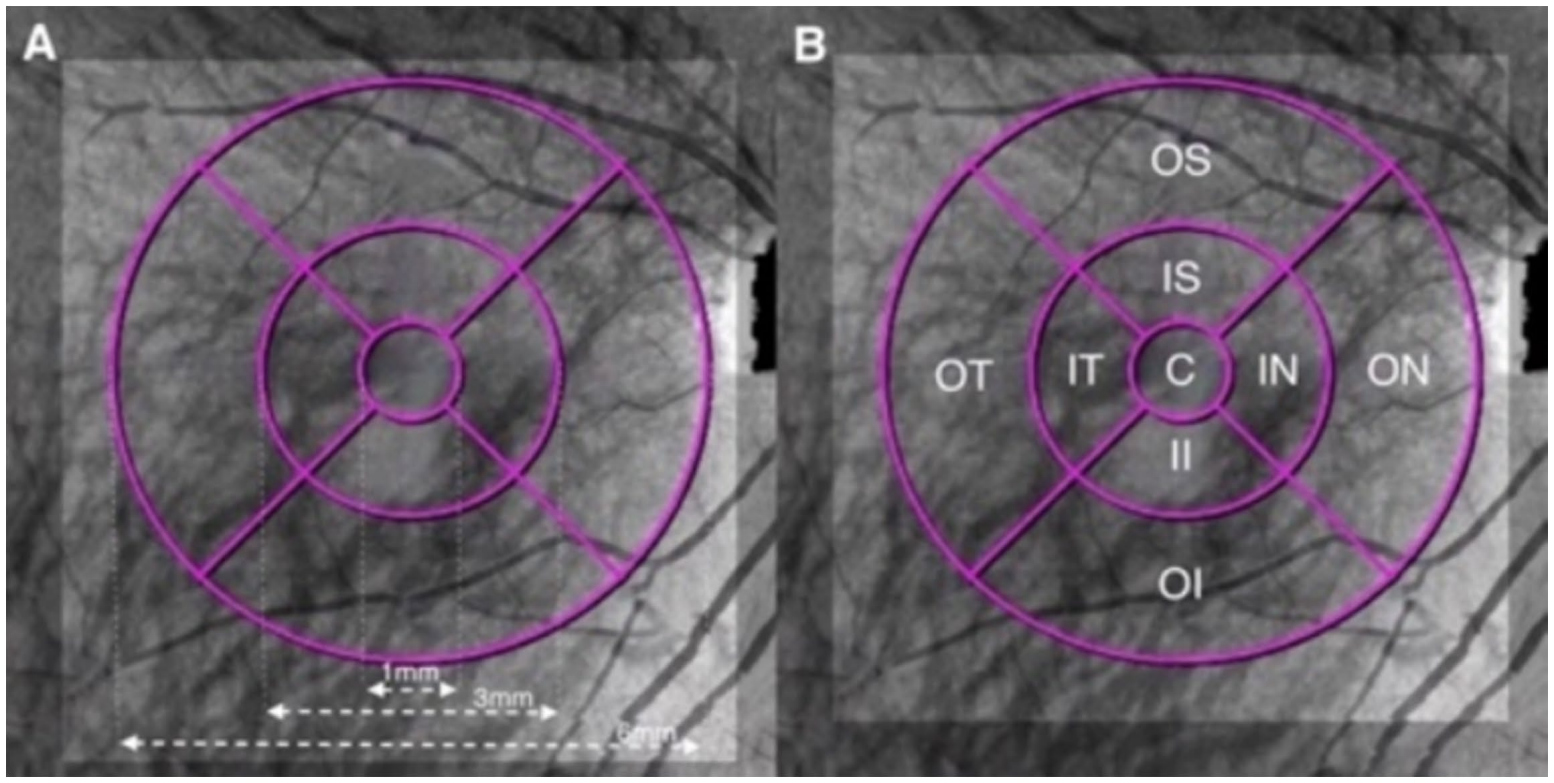
Illustration of the macular ETDRS grid of the choroid. **(A)** The macular area was divided into three concentric rings with diameters of 1, 3, and 6 mm centered on the fovea. **(B)** Quadrants: C, central fovea; IS, inner superior; IN, inner nasal; II, inner inferior; IT, inner temporal; OS, outer superior; ON, outer nasal; OI, outer inferior; and OT, outer temporal.

Based on the Early Treatment of Diabetic Retinopathy Study (ETDRS) grid, the macular zone was divided into three concentric rings with a diameter of 1 mm (central fovea, C), 3 mm (parafovea), and 6 mm (perifovea) (6). The parafoveal and perifoveal annulus were further subdivided into superior, nasal, inferior, and temporal quadrants (Figures 1, 2).

### Statistical analysis

IBM SPSS Statistics 26 (Chicago, IL, United States) software was used for statistical analysis. The Shapiro–Wilk test was performed to assess the normality of the data. Descriptive statistics were presented as means ± standard deviations unless otherwise stated. Values representing the less myopic eyes were subtracted from those of the relatively more myopic eyes to derive interocular differences. Paired *t*-tests were performed to assess binocular comparisons. Two-way repeated measures ANOVA was conducted to analyze within-subject factors (eye and region) for choroidal thickness and circulation between the fellow eyes. Pearson correlation analysis was utilized to assess the statistical significance of associations between variables, wherever appropriate. A multiple linear regression model with generalized estimating equations was used to determine the association between the interocular difference in SER and interocular differences in choroidal thickness and circulation. A *p*-value below 0.05 was deemed statistically significant.

## Results

### General ocular biometric characteristics

A total of 60 anisomyopic individuals (31 men and 29 women) with an average age of 20.03 ± 2.38 years were enrolled in this study. The general ocular biometric characteristics are summarized in Table 1. The SER of the more myopic eyes was −5.98 ± 1.72 D, while that of the relatively less myopic eyes was −3.38 ± 1.71 D (*p* < 0.001). The interocular difference in SER was −2.60 ± 1.17 D. The AL in the more myopic eyes (26.07 ± 0.86 mm) was significantly longer than that in the contralateral eyes (25.40 ± 0.23 mm) (*p* < 0.001). The interocular difference in AL was 0.67 ± 1.02 mm. The interocular difference in SER was significantly correlated with that in AL (Spearman’s correlation, R = − 0.432, *p* < 0.001). CCR, ACD, CCT, and IOP showed no differences between the paired eyes (all *p* > 0.05).

**Table 1:** Comparison of the ocular biometric parameters in anisomyopic participants.

Parameter	More myopic eyes	Less myopic eyes	Interocular difference	Paired-t	*p*-value
SER (D)	−5.98 ± 1.72	−3.38 ± 1.71	−2.60 ± 1.17	17.230	<0.001
AL (mm)	26.07 ± 0.86	25.40 ± 0.23	0.67 ± 1.02	5.090	<0.001
CCR (mm)	7.85 ± 0.28	7.92 ± 0.22	−0.07 ± 0.29	1.717	0.091
ACD (mm)	3.26 ± 0.24	3.28 ± 0.22	−0.02 ± 0.30	0.619	0.538
CCT (um)	537.78 ± 40.86	545.17 ± 32.07	−7.38 ± 44.35	1.290	0.202
IOP (mmHg)	15.17 ± 2.21	15.27 ± 2.35	−0.22 ± 3.18	0.527	0.600

SER, spherical equivalent refraction; AL, axial length; CCR, corneal curvature radius; ACD, central anterior chamber depth; CCT, corneal thickness; IOP, intraocular pressure.

### Global analysis of choroidal thickness and circulation in anisomyopia

The more myopic eyes exhibited thinner MCT (254.02 ± 50.86 vs. 292.78 ± 64.57 um) and lower CBFA (16.46 ± 1.46 vs. 17.28 ± 1.48 mm^2^), CVV (1.56 ± 1.03 vs. 2.47 ± 1.44 mm^3^), and CVI (0.19 ± 0.10 vs. 0.27 ± 0.11%) than the less myopic eyes across the whole imaging area (all *p* < 0.01). The interocular differences in MCT, CBFA, CVV, and CVI were −38.74 ± 69.66 um, −0.82 ± 1.99 mm^2^, −0.90 ± 1.55 mm^3^, and −0.08 ± 0.13%, respectively (Table 2).

**Table 2:** Comparison of the choroidal parameters in anisomyopic participants.

Parameter	More myopic eyes	Less myopic eyes	Interocular difference	Paired-t	*p*-value
MCT (um)	254.02 ± 50.86	292.78 ± 64.57	−38.74 ± 69.66	4.308	<0.001
CBFA (mm^2^)	16.46 ± 1.46	17.28 ± 1.48	−0.82 ± 1.99	3.196	<0.01
CVV (mm^3^)	1.56 ± 1.03	2.47 ± 1.44	−0.90 ± 1.55	4.509	<0.001
CVI (%)	0.19 ± 0.10	0.27 ± 0.11	−0.08 ± 0.13	4.740	<0.001

MCT, mean choroidal thickness; CBFA, choriocapillaris blood flow area; CVV, choroidal vessel volume; CVI, choroidal vessel index.

### Topographic analysis of choroidal thickness and circulation in anisomyopia

**Figure 3:**
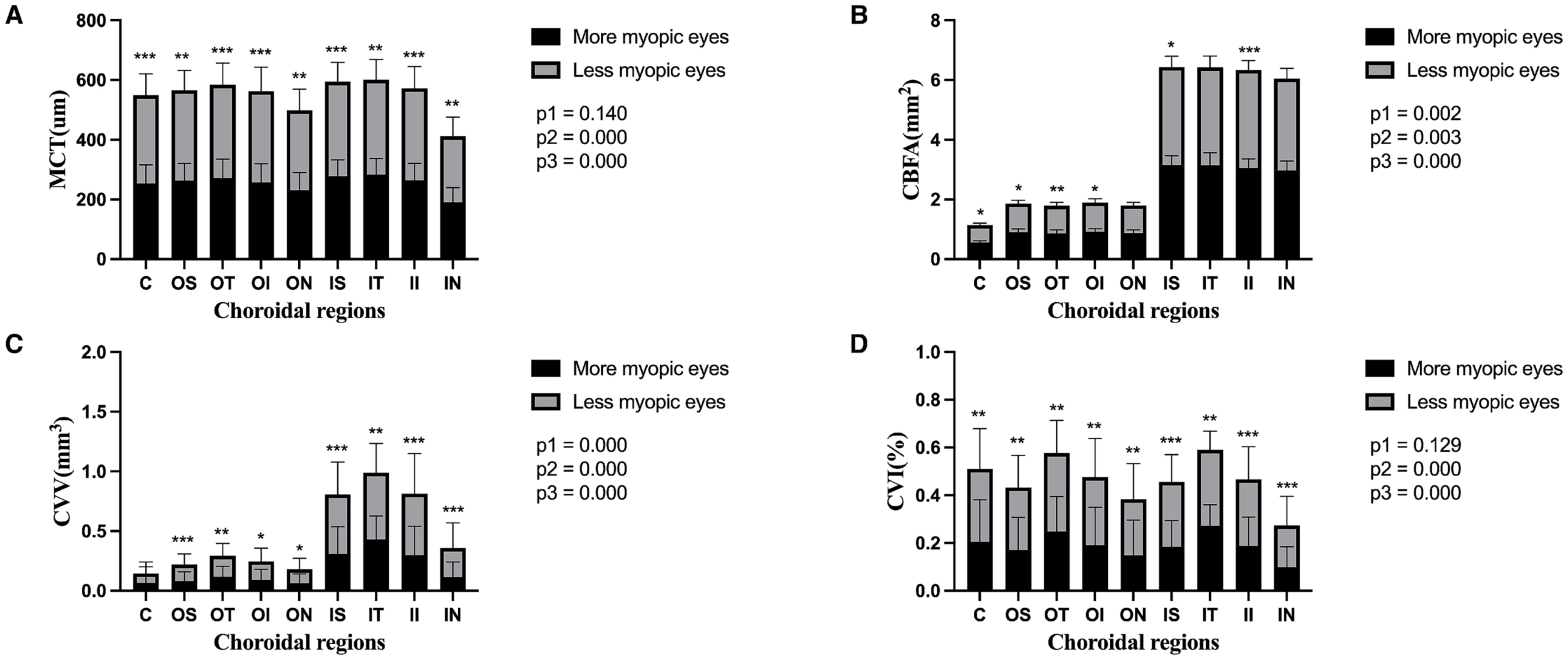
Topography of the choroidal parameters in the paired eyes of the anisomyopic individuals. Panels **(A–D)** represent MCT, CBFA, CVV, and CVI in the entire population, respectively. Data are expressed as means ± SDs. ^*^p < 0.05 and ^**^p < 0.01, ^***^p < 0.001 indicate significant differences between the paired eyes. P1, the *p*-value of the eye and region interaction; P2, the *p*-value of the main effect of the eyes; and P3, the *p*-value of the main effect of the regions.

Compared to the less myopic fellow eyes, further topographic analysis of the more myopic eyes revealed that all sub-regions had thinner MCT and lower CVI (all *p* < 0.01). Except for OS and ON sub-regions, CBFA was reduced across all the remaining regions (all *p* < 0.05), and there was also a significant decrease in CVV among all sub-regions, except the central fovea (all *p* < 0.05). The main effects of the eyes and regions on CBFA and CVV were both significant (eyes: CBFA, *F* = 9.36, *p* = 0.003; CVV, *F* = 15.03, *p* = 0.000; regions: CBFA, *F* = 239.02, *p* = 0.000; CVV, *F* = 242.60, *p* = 0.000). Furthermore, the interaction effects of the eyes and regions on CBFA and CVV were also significant (eyes × regions: CBFA, *F* = 3.14, *p* = 0.002; CVV, *F* = 10.66, *p* = 0.000). Although the main effects of the eyes and regions on MCT and CVI were both significant (eyes: MCT, *F* = 13.10, *p* = 0.000; CVI, *F* = 15.04, *p* = 0.000; regions: MCT, *F* = 201.00, *p* = 0.000; CVI, *F* = 56.63, *p* = 0.000), neither MCT nor CVI showed a significant interaction effect between the eyes and regions (eyes × regions: MCT, *F* = 1.54, *p* = 0.140; CVI, *F* = 1.57 *p* = 0.129) (Figure 3).

### Relationship between the interocular difference in SER and that in the choroidal parameters

The interocular differences in MCT, CVV, and CVI were positively correlated with that in SER (all *p* < 0.05), and the interocular differences in MCT, CVV, and CVI were negatively correlated with that in AL (all *p* < 0.05). However, there was no significant correlation between the interocular differences in SER and AL and those in CBFA (both *p* > 0.05) (Table 3).

**Table 3:** Associations between interocular differences in SER, AL, and choroidal parameters.

Parameter		MCT	CBFA	CVV	CVI
SER	R	0.314	0.166	0.278	0.258
	P	0.033	0.206	0.032	0.047
AL	R	−0.301	−0.119	−0.306	−0.278
	P	0.036	0.366	0.018	0.032

MCT, mean choroidal thickness; CBFA, choriocapillaris blood flow area; CVV, choroidal vessel volume; CVI, choroidal vessel index; SER, spherical equivalent refraction; AL, axial length. Values are Pearson’s correlation coefficients.

### Correlation between choroidal factors and the interocular difference in SER

To determine the independent choroidal factors associated with the interocular difference in SER, a generalized estimating equation was used to establish a multiple linear regression model. The final results showed that the interocular difference in SER was significantly correlated with the interocular difference in CVV (*β* = 0.209, *p* = 0.032) (Table 4).

**Table 4:** Correlation between interocular differences in choroidal parameters and interocular differences in SER.

Parameter	Unstandardized coefficient	Standardized coefficient	95% confidence interval	VIF	t	*p*-value
MCT	−0.10	−6.07	−0.023 to 0.003	8.739	−1.432	0.158
CBFA	0.57	0.10	−0.109 to 0.222	1.08	0.438	0.663
CVV	0.209	0.10	0.019 to 0.399	1.000	2.201	0.032
CVI	−2.984	−0.32	−12.563 to 6.599	16.481	−0.369	0.713

MCT, mean choroidal thickness; CBFA, choriocapillaris blood flow area; CVV, choroidal vessel volume; CVI, choroidal vessel index; VIF, variance inflation factor.

## Discussion

The relationship between the choroid and myopia remains an important topic that needs to be elucidated. Therefore, the current study explored the interocular differences in choroidal thickness and circulation in the paired eyes of anisomyopic adults, aiming to identify whether there is a potential association between the choroid and myopia. In summary, in all anisomyopic individuals, the more myopic eyes had lower MCT, CBFA, CVV, and CVI than the less myopic fellow eyes, and the interocular differences in MCT, CVV, and CVI were positively correlated with that in SER and negatively correlated with that in AL. The multiple linear regression model confirmed that the interocular difference in CVV was significantly associated with the interocular difference in SER. These results indicate that both choroidal thickness and circulation are compromised as myopia progresses, and they are more likely to decrease with worsening myopia. The choroid is regarded as an important biomarker for myopia, and our findings may shed some light on the relationship between choroid alterations and human myopia.

Recently, an inverse association between ChT and the severity of myopia has been demonstrated in some cross-sectional and longitudinal studies (20, 30, 31). Consistently, our result showed that the more myopic eyes have thinner ChT than the contralateral eyes in anisomyopic adults. Similarly, a study with a larger sample size (70 pediatric anisomyopic individuals) and another involving a smaller population (44 anisomyopic teenagers) provided evidence supporting our observations (7, 32), although the areas explored in the above two studies (3 × 3 mm) were relatively smaller than our study (6 × 6 mm). In addition, some scholars have proposed a theory named “choroidal accommodation,” pointing out that ChT has the characteristics of bidirectional variation in response to the process of imposed myopic or hyperopic defocusing (32, 33). These findings suggest that individuals with considerable ChT reduction may be more likely to experience accelerated myopic progression. However, Liu et al. (34) suggested that ChT thinning may not be solely due to passive stretching from axial myopia, and Vincent et al. (35) also suggested that variation in globe expansion from simple passive stretching cannot fully account for the binocular difference in ChT observed between the paired eyes of anisomyopic individuals. Therefore, it is reasonable to speculate that changes in ChT may be related to the modulation of myopic eye elongation to some extent, but additional factors must also be considered.

Anatomically, based on the highly vascularized choroid and its characteristics of rapidly changing blood flow, it seems plausible that variation in choroidal circulation may primarily contribute to changes in ChT. Currently, several pharmacological studies have demonstrated that changes in ChT are accompanied by concomitant variation in choroidal perfusion, with both parameters showing enhancement after administration of prazosin (a vasodilator) (36) and a decrease after intravitreal injection of bevacizumab (an anti-vascular endothelial growth factor drug) (37). Moreover, this feature of bidirectional changes in choroidal circulation has also been observed in myopic models, with decreases during experimentally induced myopia and increases during subsequent recovery (38). Therefore, considering that choroidal thinning may reflect myopia development, it is plausible to theorize that myopic-related ChT reduction might coincide with diminished choroidal circulation. Furthermore, it is conceivable that choroidal circulation may be directly or indirectly related to myopia, with or without the involvement of ChT.

Our main finding was that choroidal vascularity was lower in the more myopic eyes of the anisomyopic individuals. The concomitant decrease in CVV and CVI revealed that the large and middle choroidal vessels gradually deteriorate with increasing myopia, suggesting a potential connection between choroidal vascularity attenuation and myopia. Our results are in line with several recent studies. Gupta et al. (39) first reported a reduction in choroidal vascular area in patients with high myopia. Then, Li et al. (40) reported that the luminal area of the choroid exhibits a decreasing trend in low-to-moderate myopia. Later, Wu et al. (6) and Su et al. (41) demonstrated that reduction in choroidal vascularity was positively associated with increased myopic severity. More recently, a study on pediatric anisomyopic patients reported that the more myopic eyes exhibited reduced choroidal vascularity than the fellow eyes (7). Collectively, all these findings indicate that decreased choroidal vascularity is associated with myopia progression. A key issue that remains unresolved is whether attenuation in choroidal vascularity leads to myopia development or vice versa; their cause-and-effect relationship needs to be illustrated.

Considering that the choriocapillaris is supplied by the large and middle vascular tissues, variations in choroidal vascularity may affect its circulation, but the existing conclusions are inconsistent. Milani (42) and Liu et al. (32) found no significant differences in choriocapillaris perfusion between the control and myopic eyes. Similarly, Scherm et al. (43) observed that choriocapillaris blood flow remained stable with increasing physiological myopia. In contrast, in both anisomyopic adults and children, Wu et al. (6, 7) discovered that the eyes with a greater degree of myopia tended to have higher choriocapillaris flow deficits than the contralateral eyes. Moreover, Al-Sheikh et al. (44) studied patients with high myopia and confirmed that choriocapillaris flow deficits had an increasing tendency in eyes with severe myopia. However, Mo et al. (45) reported that choriocapillaris flow densities decreased in pathological myopia. Our result revealed a significant decrease in CBFA in the more myopic eyes. Different sizes of covered choroidal regions, variations in detection indicators that identify choriocapillaris perfusion, and, more importantly, the lack of unified and standardized machine algorithms may largely account for the inconsistent results.

Recently, hypoxia has emerged as a new perspective. Hao et al. (1) first creatively put forward a new theory of myopia: External visual stimulation may change hemodynamics and hemorheology by regulating choroidal circulation, thereby causing hypoxia in the adjacent scleral, which, in turn, modulates scleral remodeling and axial elongation, eventually leading to the development of myopia. This theory provides a new insight into the role of the choroid in the pathogenesis of myopia. Later, their another study revealed that increased choroidal perfusion can inhibit both the development of myopia and the elongation of the eyeball by attenuating scleral hypoxia (36). Consistently, our results showed that the more myopic eyes were associated with thinner ChT and reduced choroidal circulation. Therefore, it is plausible that reduced ChT and circulation may result in a relatively hypoxic environment, which could trigger multiple downstream receptor-related signaling pathway events, and, in turn, induce responses to promote the onset and progression of myopia.

There are limitations that should not be overlooked. First, our study consisted of a relatively small population and included only young subjects. Further research is needed with larger populations across different age groups, and long-term, longitudinal observations are warranted. In addition, whether a causal relationship exists between choroidal thickness and circulation and whether these two parameters have correlated or independent effects on the onset and development of myopia remain to be illustrated. Finally, we did not consider some important confounding factors, such as ocular perfusion pressure and systolic blood pressure.

## Conclusion

In conclusion, both choroidal thickness and circulation were found to be reduced in the more myopic eyes of anisomyopic adults. We observed that the greater the interocular difference in SER, the lower the choroidal circulation and thinner thickness, indicating that the choroid is affected in individuals with myopia and is more likely to be compromised in severe myopia. Although current evidence remains insufficient to make any definitive conclusions regarding the association between the choroid and myopia, our findings may contribute to a better understanding of their relationship.

## Data Availability

The raw data supporting the conclusions of this article will be made available by the authors, without undue reservation.
